# Diagnostic Methods, Clinical Guidelines, and Antibiotic Treatment for Group A Streptococcal Pharyngitis: A Narrative Review

**DOI:** 10.3389/fcimb.2020.563627

**Published:** 2020-10-15

**Authors:** Zahid Mustafa, Masoumeh Ghaffari

**Affiliations:** Department of Internal Medicine, University of California, Riverside, Riverside, CA, United States

**Keywords:** group A strep, group A b-hemolytic streptococcus****, group A streptococcus, group A streptococcal pharyngitis, pharyngitis, sore throat, strep throat

## Abstract

The most common bacterial cause of pharyngitis is infection by Group A *β*-hemolytic streptococcus (GABHS), commonly known as strep throat. 5–15% of adults and 15–35% of children in the United States with pharyngitis have a GABHS infection. The symptoms of GABHS overlap with non-GABHS and viral causes of acute pharyngitis, complicating the problem of diagnosis. A careful physical examination and patient history is the starting point for diagnosing GABHS. After a physical examination and patient history is completed, five types of diagnostic methods can be used to ascertain the presence of a GABHS infection: clinical scoring systems, rapid antigen detection tests, throat culture, nucleic acid amplification tests, and machine learning and artificial intelligence. Clinical guidelines developed by professional associations can help medical professionals choose among available techniques to diagnose strep throat. However, guidelines for diagnosing GABHS created by the American and European professional associations vary significantly, and there is substantial evidence that most physicians do not follow any published guidelines. Treatment for GABHS using analgesics, antipyretics, and antibiotics seeks to provide symptom relief, shorten the duration of illness, prevent nonsuppurative and suppurative complications, and decrease the risk of contagion, while minimizing the unnecessary use of antibiotics. There is broad agreement that antibiotics with narrow spectrums of activity are appropriate for treating strep throat. But whether and when patients should be treated with antibiotics for GABHS remains a controversial question. There is no clearly superior management strategy for strep throat, as significant controversy exists regarding the best methods to diagnose GABHS and under what conditions antibiotics should be prescribed.

## Introduction

Pharyngitis is one of the most common reasons people seek medical care. 1–2% of visits to physicians’ offices and emergency departments in the United States every year are for sore throat (Prevention CfDCa, 2016a; Prevention CfDCa, 2017). Estimates of the number of Americans who seek care for pharyngitis annually vary from 11 to 18 million (Prevention CfDCa, 2008; Prevention CfDCa, 2016b). As many as four to six times more individuals suffer from sore throat and elect not to seek care (Neuner et al., 2003).

Pharyngitis is caused by many different types of infectious agents. The majority of cases have viral etiologies. The most common bacterial cause of pharyngitis is infection by Group A *β*-hemolytic streptococcus (GABHS), commonly known as strep throat. There are more than 616 million new cases of GABHS worldwide each year (Carapetis et al., 2005). 5–15% of adults and 15–35% of children in the United States with pharyngitis have a GABHS infection (Cooper et al., 2001; Linder and Stafford, 2001; Linder et al., 2005; Shulman et al., 2012; Stewart et al., 2014). The economic burden associated with GABHS is substantial. In the United States the cost of diagnosing, treating, and caring for children with strep throat is between $224 and $539 million per year (Pfoh et al., 2008).

Symptoms of GABHS include throat pain, fever, headaches, and chills. Other possible indications are abdominal pain, nausea, and vomiting. Individuals with strep throat typically do not present with a cough, coryza, or conjunctivitis (Wessels, 2011). Left untreated GABHS may lead to nonsuppurative and suppurative complications like acute rheumatic fever, rheumatic heart disease, poststreptococcal glomerulonephritis, bacteremia, peritonsillar abscess, and retropharyngeal abscess (Ebell et al., 2000). Unfortunately, the symptoms of GABHS overlap quite broadly with viral etiologies, complicating the problem of diagnosis.

Transmission of GABHS results from contact with respiratory tract secretions of infected individuals (Langlois and Andreae, 2011). The incubation period is 2–5 days. During this time the infection can be transmitted to others (Snow et al., 2001). GABHS is treatable through administration of appropriate antibiotics. Treatment reduces the period of communicability to 24 h in 80% of cases (Van Brusselen et al., 2014).

The purpose of this article is to provide a comprehensive overview of the current state of knowledge pertaining to GABHS. Existing reviews are focused on particular aspects of the disease: clinical scoring systems for children (Shaikh et al., 2012; Le Marechal et al., 2013; Cohen J. F. et al., 2015), clinical scoring systems for adults (Aalbers et al., 2011), international guidelines for diagnosing and treating pharyngitis (Matthys et al., 2007; Chiappini et al., 2011; Van Brusselen et al., 2014), rapid antigen detection tests (RADTs) for GABHS (Gerber and Shulman, 2004; Stewart et al., 2014; Cohen et al., 2016), and antibiotic treatment (Altamimi et al., 2012; Spinks et al., 2013; van Driel et al., 2016). In this review we cover methods for diagnosing the infection, clinical guidelines for strep throat, and the question of treatment. Conducting a broad review of GABHS provides physicians information useful for addressing the different aspects of caring for patients with pharyngitis, while highlighting gaps in knowledge researchers should address.

## Diagnostic Methods for GABHS

GABHS manifests as throat pain, fever, headaches, and chills (Wessels, 2011). These symptoms overlap with non-GABHS and viral causes of acute pharyngitis. Therefore, obtaining an accurate differential diagnosis based solely on a patient’s symptoms is difficult. A careful physical examination and patient history is the starting point for diagnosing GABHS. Diagnosis of GABHS over the phone is not advised, as the pharynx and cervical lymph nodes cannot be examined (Sheridan et al., 2007). Furthermore, patients have difficulty in accurately measuring the severity of their symptoms, creating a positive diagnostic bias (Xu et al., 2004).

After a physical examination and patient history is completed, five types of diagnostic methods can be used to ascertain the presence of a GABHS infection. The first is clinical scoring systems. The second is rapid antigen detection tests. The third is throat culture. The fourth is nucleic acid amplification tests. The fifth is machine learning and artificial intelligence.

### Clinical Scoring Systems

No single symptom has sufficient diagnostic accuracy to confirm or rule out the presence of GABHS (Aalbers et al., 2011). A number of different clinical scoring systems have been proposed to help physicians diagnose strep throat. These algorithms integrate information from different variables to assess the probability that a patient has GABHS. The most popular scoring system is the Centor criteria. The Centor scoring system assigns one point for each of four symptoms (**Table 1**). These points are then added to yield a composite score. Higher scores indicate greater likelihood of GABHS. The Centor algorithm was derived from a sample of adults visiting an urban emergency room. In this sample individuals with a maximum score of four had a 56% probability of a positive GABHS culture, while those with the minimum score of zero had just a 2.5% probability of a positive GABHS culture (Centor et al., 1981).

**Table 1:** Comparison of Centor *vs.* McIsaac Scoring Systems.

Centor Symptoms (1981)	Point	McIsaac Symptoms (1998)	Point
**Tonsillar exudates**	1	**Tonsillar swelling or exudate**	1
**Swollen tender anterior cervical nodes**	1	**Tender anterior cervical adenopathy**	1
**Lack of cough**	1	**No cough**	1
**Fever history**	1	**Temperature > 38°C**	1
		**Age 3–14 years**	1
		**Age 15–44 years**	0
		**Age ≥ 45 years**	-1
**Total score**		**Total score**	

The McIsaac scoring system modifies the Centor criteria by using age to account for the increased prevalence of GABHS in children (**Table 1**). Like the Centor algorithm, a higher McIsaac composite score means greater risk of a GABHS infection. In the study that produced the scoring system, the sensitivity (the proportion of GABHS-positive individuals who are identified as such) of the McIsaac algorithm was 83.1%. The sensitivity of the McIsaac scoring system was higher than the 69.4% achieved using physicians’ clinical judgment based on an encounter form and a physical exam. Specificity (the proportion of GABHS-negative individuals who are identified as such) between the two methods was similar, at 94.3% for the McIsaac criteria and 96.6% for physician’s standard clinical judgment (McIsaac et al., 1998).

A number of other clinical scoring systems have been proposed to guide physicians. One algorithm uses a combination of seven physical symptoms and historical features to facilitate diagnosis (Komaroff et al., 1986). Another approach applies a Bayesian framework to derive fourteen different variables that can be used to predict a GABHS infection (Dobbs, 1996). FeverPAIN is a five-item prediction rule that integrates information on whether an individual has experienced fever during the previous 24 h, is purulent, attends rapidly, has inflamed tonsils, and does not exhibit cough or coryza (Little et al., 2013). The Walsh diagnostic algorithm provides a decision analysis tool to classify people as either at low, moderate, or high risk of having strep throat (Walsh et al., 1975). Breese offers a nine-factor scorecard for assessing the likelihood a person has GABHS (Breese, 1977). Many scoring systems have also been proposed specifically for children (Le Marechal et al., 2013), including one specifically designed for use in areas where cost considerations preclude use of biologic testing (Joachim et al., 2010).

The number of studies that have proposed different clinical scoring systems show that there is intense interest in accurate diagnosis of GABHS. Alternatives to the Centor and McIsaac algorithms are not widely used, most likely due to the difficulty of implementing their more complex criteria. Attention has thus focused on validating the Centor and McIsaac scoring systems. There is significant disagreement on the merits of these systems for diagnosing GABHS. Some studies assert that the Centor algorithm has been validated in different settings and demonstrates reasonable sensitivity and high specificity (Wigton et al., 1986; Neuner et al., 2003; Sheridan et al., 2007; Aalbers et al., 2011; Fine et al., 2012). McIsaac and colleagues validated the modified McIsaac criteria in a new sample and found that the scoring system is both accurate and reliable for diagnosing GABHS in adults and children (McIsaac et al., 2000).

However, several meta-analyses and systematic reviews argue that the Centor and McIsaac algorithms are not sufficient to establish a diagnosis of GABHS, particularly in children. A meta-analysis of sixteen decision rules for children concludes that most validation studies have serious methodological flaws, contributing to low positive and negative predictive values (Le Marechal et al., 2013). The authors of a systematic review reached a similar conclusion: the Centor scoring system alone cannot produce a definitive diagnosis in children (Shaikh et al., 2012). Possible reasons for the poor performance of decision rules in children are that only children with severe symptoms are brought in for evaluation—in a sense “using up” the diagnostic value of the rules—or the higher incidence in children of viral infections showing the same symptoms of fever, sore throat, and enlarged lymph nodes (Stefaniuk et al., 2017).

Whether scoring systems by themselves provide an adequate basis to diagnose GABHS remains an open question. While acknowledging this uncertainty, the Centor and McIsaac algorithms have two advantages that make them attractive tools for physicians. The first is that they do not require specialized equipment and are easy for providers to implement. The second is that scoring systems can be used to focus on other diagnostic methods. Doing so helps limit the number of false positives that will occur if all patients are given a biologic test, as tests without clinical assessment identify asymptomatic GABHS carriers as patients with an active infection (Felsenstein et al., 2014; Tanz et al., 2019).

### Rapid Antigen Detection Tests (RADTs)

RADTs have been used for four decades to help physicians diagnose GABHS. There are three principal types of RADTs: latex agglutination, enzyme immunoassay (EIA), and optical immunoassay (OIA) (Cohen et al., 2016). All rapid tests begin with a throat swab. Acid extraction is then used to solubilize GABHS cell wall carbohydrate. An immunological reaction detects the presence or absence of the Lancefield group A carbohydrate, a GABHS-specific cell-wall antigen (Gerber and Shulman, 2004; Chiappini et al., 2011; Cohen et al., 2016). Results are available in fewer than 10 min. The process does not require specially trained personnel.

A number of commercially-available RADTs exist. These tests show three characteristics. First, the sensitivity of RADTs is generally lower than specificity (**Table 2**). Systematic reviews and meta-analyses provide a point estimate of RADT sensitivity of approximately 85%, and specificity of approximately 96% (Ruiz-Aragon et al., 2010; Lean et al., 2014; Cohen et al., 2016). Second, rapid tests vary widely in sensitivity and specificity. Review studies and clinical guidelines report varying ranges (**Table 3**). And third, validation tests provide evidence that sensitivity in the clinical environment may be significantly lower than suggested by manufacturers (Forward et al., 2006).

**Table 2:** Sensitivity and Specificity of Select Rapid Antigen Detection Tests.

RADT	Sensitivity (%)	Specificity (%)	Source
**Acceava Strep A**	94.0	Not reported	Nakhoul and Hickner (2013)
	98.5	98.8	Rogo et al. (2011)
**QuikRead go^®^ Strep A**	85.0	91.0	Stefaniuk et al. (2017)
	80.0	73.3	Azrad et al. (2019)
**Sofia StrepA FIA**	84.9	96.8	Lacroix et al. (2018)
**Genzyme OSOM Strep A**	98.5	99.4	Rogo et al. (2011)
**Alere TestPack Strep A**	75.3	98.1	Lacroix et al. (2018)
**Strep A Rapid Test Device**	71.9	94.3	Forward et al. (2006)
**Strep A OIA MAX**	94.7	100.0	Ezike et al. (2005)
	92.4	96.3	Ezike et al. (2005)
**Quidel QuickVue Strep A**	69.6	97.8	Tanz et al. (2009)
	92.3	96.3	Rogo et al. (2011)
	100.0	98.8	Safizadeh Shabestari et al. (2019)
**Abbott TestPack Strep A Assay**	79.4	100.0	Heiter and Bourbeau (1995)
**BD Veritor™ System**	80.0	78.7	Azrad et al. (2019)
**BioStar Strep A OIA**	87.1	97.4	Needham et al. (1998)
	97.4	95.6	Harbeck et al. (1993)
	98.9	98.6	Harbeck et al. (1993)
	84.2	95.7	Daly et al. (1994)
	91.5	94.8	Heiter and Bourbeau (1995)
	84.0	93.0	Gerber et al. (1997)

**Table 3:** Sensitivity and Specificity Ranges and Point Estimates for RADTs Reported in Select Review Studies.

Review	Sensitivity Range (%)	Sensitivity Point Estimate (%)	Specificity Range (%)	Specificity Point Estimate (%)
Snow et al. (2001)	58–96	Not Reported	63–100	Not Reported
Gerber and Shulman (2004)	70–90	Not Reported	95–100	Not Reported
Le Marechal et al. (2013)	85–90	Not Reported	90–100	Not Reported
Webb (1998)	77–97	89	89–99	95
Neuner et al. (2003)	70–99	88	80–99	94
Lean et al. (2014) (OIA only)	71–95	86	86–100	96
Cohen et al. (2016) (children only)	39–100	86	54–100	95
Ruiz-Aragon et al. (2010)	66–96	85	69–99	96

There are a number of possible explanations for the heterogeneity observed in RADT sensitivity and specificity. Some assessments of RADT performance are conducted in clinical settings, while others are research studies conducted by trained staff. The methods tests use to determine the presence of GABHS also differ, another source of variance. Lack of consistency in how studies report RADT results can increase heterogeneity for tests that have similar sensitivity and specificity (Stewart et al., 2014). Whether throat swabs are performed on the posterior pharynx and tonsils as opposed to the tongue, lips, and buccal mucosa affects sensitivity, although the extent of variation caused by differences in sample location remains a matter of dispute (Wessels, 2011; Adler et al., 2020). The experience of the person performing the RADT also matters, as does the absence of a universally accepted blood agar plate culture method to serve as a reference standard (Gerber and Shulman, 2004). Patient-level characteristics like clinical presentation and inoculum size, and physician-level attributes like whether a doctor practices in a hospital or an office have been found to affect sensitivity (Cohen et al., 2013). Finally, it is possible that the sensitivity of a given RADT is not fixed but is a function of the severity of the disease or the likelihood a patient is infected, a phenomenon known as spectrum bias (Dimatteo et al., 2001; Hall et al., 2004; Plainvert et al., 2015).

RADTs have three attributes that make them useful clinical tools. The first is that they provide quick results to inform diagnosis and treatment. The second is that they are inexpensive; many test kits are available for between $1 and $2 per sample. The third is that they are simple to use; middle school students with limited training have demonstrated an ability to perform RADTs (Gerber and Shulman, 2004). Given these beneficial properties it is surprising that fewer than fifty percent of physicians use RADTs to assist in diagnosis of GABHS (Le Marechal et al., 2013; Teratani et al., 2019). The high reported variance of different RADTs may discourage physicians from employing them. Another possibility is that when the percentage of the population infected with GABHS is low, RADTs require a high sensitivity to avoid false negatives (Forward et al., 2006). RADTs also fail to distinguish between the carrier state and an active infection, although combining their use with a physical examination should minimize false positives. Given the absence of clear standards on what constitutes acceptable sensitivity and specificity clinicians are left to make their own determination on whether to use a specific RADT to assist in diagnosing GABHS (Gerber and Shulman, 2004).

### Throat Culture

Throat culture is considered to be the reference standard for diagnosing GABHS. Swabs of the posterior pharynges and tonsils are taken and then cultured, typically on a 5% sheep-blood agar plate. GABHS identification is performed based on colony morphology, Gram stain, and serogrouping (Henson et al., 2013). The principal advantages of throat culture for diagnosing GABHS are its high sensitivity and specificity and its low cost (Neuner et al., 2003). Other benefits are the possibility of identifying other pathogens that can cause pharyngitis and enabling antibiotic susceptibility testing. The main disadvantage of throat culture is that it takes 24 to 48 h to obtain test results, delaying diagnosis and treatment. A secondary disadvantage is the inability of culturing to distinguish on its own between an acute infection and a carrier state, producing false positives (Snow et al., 2001; Aalbers et al., 2011). Although throat culture is considered to be the reference standard for diagnosing GABHS, it does not have perfect sensitivity and specificity. Test–retest trials do not always concur, results do not always correlate with other high-sensitivity and specificity methods like PCR and antibody titers, and differences in culturing technique yield differing outcomes (Snow et al., 2001; Neuner et al., 2003; Anderson et al., 2013).

### Nucleic Acid Amplification Techniques (NAATs)

NAATs also exist to diagnose GABHS. Probe testing detects nucleic acid sequences that are specific to GABHS (*e.g*. from the speB or sda genes). Although NAATs have higher sensitivity than RADTs, their high cost (one popular product has a list price of $69.50 per test) precludes widespread use as a replacement for throat culture (Nakhoul and Hickner, 2013; Weinzierl et al., 2018). A number of NAATs have received FDA clearance over the past six years (Luo et al., 2019). One such test is the *illumi*gene assay, a diagnostic test that works through a loop-mediated isothermal amplification process. The *illumi*gene assay has high sensitivity and specificity: estimates of 99.0% sensitivity and 99.6% specificity (Anderson et al., 2013) and 100% sensitivity and 99.2% specificity have been reported (Henson et al., 2013), indicating that the assay can be a useful diagnostic tool for GABHS (Felsenstein et al., 2014). Studies of the LightCycler Strep-A assay and the Xpert Xpress Strep A test report sensitivities of 93 and 100% and specificities of 98 and 79%, respectively (Uhl et al., 2003; Ralph et al., 2019). Another NAAT is the Alere i strep A test, which has sensitivity of 98.7% and specificity of 98.5%, and can be completed in just 8 min (Cohen D. M. et al., 2015). The cobas Liat strep A assay takes 15 min to yield results. According to one study of eighty-four specimens, this test has sensitivity of 100.0% and specificity of 98.3% (Uhl and Patel, 2016). An analysis of the system deployed in five primary care clinics produced similar results (Wang et al., 2017). NAATs like the Alere i strep A test and the cobas Liat strep A assay combine high sensitivity and specificity with speed, making these technologies promising candidates for point-of-care use in the clinical environment (Cohen D. M. et al., 2015; Uhl and Patel, 2016; Donato et al., 2019). Further clinical studies will be required to determine the value of NAATs for diagnosing GABHS, as most of the aforementioned studies were conducted in laboratory settings. Like RADTs and throat culture, NAATs cannot by themselves discriminate between an infection and a carrier state. Therefore, they must be supplemented with a physical examination to avoid negatively affecting antimicrobial stewardship efforts (Tanz et al., 2019).

### Machine Learning and Artificial Intelligence

In the past few years machine learning and artificial intelligence techniques have been proposed to help physicians diagnose strep throat. One novel approach uses the camera and flashlight built into a smartphone to take a picture of a patient’s throat. An add-on device is attached to the smartphone to minimize the reflection of light into the camera sensor. Image correction algorithms are then implemented, and *k*-fold validation applied for classification. Experimental results from a sample of 28 healthy and 28 strep-positive subjects show that the image processing method has specificity of 88% and sensitivity of 88% (Askarian et al., 2019). Given the small sample-size this approach will require validation in future studies. Another method that has been suggested is to program neural networks to assist diagnosis. One study using data from thirty-eight variables contained in 240 patients’ medical records found that a neural network can correctly diagnose pharyngitis in 95.4% of cases (Farhan S and Mahafza, 2015). Whether this promising result can be replicated in diagnosing GABHS should be examined. Artificial intelligence software has also been employed to automate the process of examining throat cultures to identify GABHS. Automatic detection of GABHS produces results that are superior to classification decisions made by lab technicians, improving diagnostic accuracy (Van et al., 2019).

## Clinical Guidelines for GABHS

How should physicians choose among the many different methods available to diagnose GABHS? Guidelines developed by professional associations can help medical professionals choose among available techniques. One set of guidelines is endorsed by the Centers for Disease Control (CDC), American Academy of Family Physicians (AAFP), and the

American College of Physicians–American Society of Internal Medicine (ACP-ASIM). Published in 2001, these guidelines recommend combining the Centor clinical decision rule with RADTs to diagnose GABHS. No testing is suggested for patients with Centor scores from zero to one. For individuals with scores from two to four the guidelines offer three options: test patients using RADTs and treat those with positive results, test patients with scores from two to three and treat those with positive tests and scores of four, and empirical treatment of patients with scores from three to four. The guidelines assert that no backup throat testing for negative RADTs is required if the sensitivity of the tests exceeds 80% (Cooper et al., 2001).

The CDC/AAFP/ACP-ASIM guidelines are controversial because they give physicians the option to diagnose and treat GABHS solely using a clinical decision rule. Some studies argue that doing so is not prudent and will cause antibiotics to be prescribed to individuals who are not infected with GABHS (Bisno, 2003; Linder et al., 2006; Shaikh et al., 2012). One research team estimated that diagnosing GABHS without a RADT may lead to more than 40% of adult patients being prescribed antibiotics unnecessarily (McIsaac et al., 2004). An investigation of pediatrician behavior reported that antibiotic prescription rates for children fell by forty-two percentage points after doctors were given the results of RADTs (Kose et al., 2016). These results have been confirmed in randomized control trials of RADTs, which conclude that physicians who use RADTs prescribe antibiotics at lower rates than physicians who do not (Worrall et al., 2007; Llor et al., 2011; Cohen et al., 2020). Modeling techniques that compare different diagnostic methods support these findings, determining that empirical treatment is neither the most effective nor the least expensive technique when the percentage of individuals presenting with pharyngitis is less than 70% (Neuner et al., 2003).

The Infectious Diseases Society of America (IDSA) issued guidelines in 2012 for diagnosing GABHS. These guidelines do not endorse the use of clinical decision rules as the sole means to diagnose GABHS. According to the IDSA, RADTs and/or throat culture should be performed because clinical features cannot differentiate GABHS from viral pharyngitis on their own. IDSA recommends performing a throat culture for children and adolescents with negative RADT results, but not in adults due to the low incidence and low risk of subsequent rheumatic fever in this population (Shulman et al., 2012). Excluding routine backup throat cultures in adults with negative RADT results has been criticized in a study that validated IDSA guidelines. According to the authors of the report, doing so will result in a false negative rate of 25% (McIsaac et al., 2004).

European physicians place less emphasis on diagnosing GABHS than their American counterparts. The European Society for Clinical Microbiology and Infectious Diseases (ESCMID) 2012 guidelines state that RADTs are unnecessary for patients with Centor scores from zero to two, but can be considered for those with Centor scores from three to four. Routine backup throat culture for individuals with negative RADT results is not recommended. The guidelines also assert that the diagnostic value of the Centor system is lower in children than in adults, due to differences in the clinical presentation of GABHS infection in children (Group et al., 2012). A study that included six different European national guidelines notes that European guidelines do not explicitly recommend using the Centor system or throat culture to diagnose GABHS. There is also a tendency in European guidelines to advise against using RADTs due to their modest sensitivities and inability to distinguish between carriers and individuals with an active infection (Matthys et al., 2007). The lack of importance assigned to diagnostic methods for GABHS evident in European guidelines reflect a view that strep throat is a self-limiting disease which will resolve without intervention in nearly all cases.

Guidelines for diagnosing GABHS created by American and European professional associations vary significantly (Chiappini et al., 2011). Areas of disagreement include whether a physical exam and clinical decision rules are sufficient to make a diagnosis, situations in which RADTs should be used, and the need for a backup throat culture of negative RADT results. These differences are the result of how physicians interpret the relevant literature, and the costs and weights assigned to possible treatments and outcomes. Discrepancies in guidelines should be reduced by employing a standardized guideline development method, a process endorsed by the World Health Organization (Matthys et al., 2007). Improvements in diagnostic tools over the past decade and the emergence of new diagnostic methods warrant review and updating of previously issued clinical guidelines. Using a standardized guideline development method should harmonize physicians and other health professionals’ approaches to diagnosing and treating GABHS, reducing confusion and leading to better health outcomes.

Determining appropriate guidelines will matter little if physicians do not use them. There is substantial evidence that most physicians do not follow any published guidelines for diagnosing and treating pharyngitis. Less than 1% of physicians explicitly document using a clinical decision rule in their exam notes (Linder et al., 2006), and a study that interviewed forty general practitioners found that not a single physician in the sample explicitly referenced the Centor criteria (Kumar et al., 2003). Attempts to induce clinicians to use the Centor score to diagnose GABHS have not been successful (Aalbers et al., 2011). There is also no relationship between Centor scores and whether a patient is given a RADT or throat culture, in part because physicians administer tests to patients using the guidelines they consider to be at low risk of being GABHS-positive (Linder et al., 2006). Studies of a large retail health chain and an emergency department note the existence of “institutional policies” requiring the use of the Centor and McIsaac scores for cases of acute pharyngitis, suggesting that practice setting plays a role in guideline adherence (Fine et al., 2012; Felsenstein et al., 2014). Future research should examine this possibility, and explore how to encourage physicians to use guidelines so that their diagnoses of GABHS are more consistent and more accurate.

## Treatment for GABHS

Treatment for GABHS has five goals. The first is to provide symptom relief. The second is to shorten the duration of illness. The third is to prevent nonsuppurative and suppurative complications. The fourth is to decrease the risk of contagion. The fifth is to decrease unnecessary use of antibiotics, slowing the development of antibiotic resistance.

Symptom relief for GABHS is straightforward and can be achieved through the use of analgesic and antipyretic agents like acetaminophen (Shulman et al., 2012). Appropriate antibiotics reduce the duration of illness by approximately one day (Sheridan et al., 2007), with the greatest reduction in symptoms seen on the third day of treatment (Spinks et al., 2013). Improvement in symptoms may depend on the speed with which antibiotics are administered. Several studies note that treatment within 48 h of the onset of symptoms provides the best chance of relief (Cooper et al., 2001; Snow et al., 2001).

Rheumatic fever is the most common nonsuppurative complication of GABHS. However, the incidence of rheumatic fever is low in the United States and other high-income countries (Snow et al., 2001; Gerber et al., 2009). Antibiotics may decrease the incidence of suppurative complications like peritonsillar abscesses (Cooper et al., 2001; Sheridan et al., 2007). The benefits of antibiotics for preventing peritonsillar abscesses are limited when patients do not present until the complication has developed (Cooper et al., 2001). The rate of transmission for individuals infected with GABHS is approximately 35% (Langlois and Andreae, 2011). Antibiotics reduce the communicability of GABHS to 24 h, and aim to limit the spread of GABHS for high-risk patients (Matthys et al., 2007).

The benefits of antibiotic therapy for achieving these objectives must be weighed against the costs of antibiotic treatment. There is clear evidence that antibiotics are overprescribed to treat GABHS, a behavior prevalent in all medical specialties (Nyquist et al., 1998). Estimates of the rate of antibiotic prescription for American adults who seek treatment for pharyngitis vary: researchers have reported values of 75 (Neuner et al., 2003), 47 (Linder et al., 2006), 70 (Linder and Stafford, 2001), and 73% (Nakhoul and Hickner, 2013). A nationwide study also found that physicians prescribe antibiotics to children with sore throat 53% of the time (Linder et al., 2005). Overuse of antibiotics may be an even greater problem in low and middle-income countries. Analysis of physician practices at three hospitals in Egypt found that doctors prescribed antibiotics to 86% of patients with pharyngitis (Ahmed MH et al., 2015).

The large discrepancy between the prevalence of GABHS in patients with pharyngitis and antibiotic prescription rates increases medical costs and creates risks for patients. Wasteful financial expenses are incurred when individuals who are not infected with GABHS are prescribed antibiotics (Humair et al., 2006). People who receive antibiotics unnecessarily may also experience adverse effects like allergic reactions and diarrhea (Neuner et al., 2003; Humair et al., 2006). Widespread use of antibiotics to treat GABHS is also causing resistance to occur in broad-spectrum macrolides and fluoroquinolones (Linder and Stafford, 2001; Neuner et al., 2003).

If antibiotics are to be used for GABHS, which antibiotics should physicians prescribe? There is broad agreement that antibiotics with narrow spectrums of activity are appropriate for treating GABHS. Penicillin V is the first-choice antibiotic of many physicians and is endorsed by CDC/AAFP/ACP-ASIM guidelines. GABHS has no known resistance to penicillin (Linder and Stafford, 2001) and an allergic reaction rate that is less than 4% (Neuner et al., 2003). IDSA guidelines recommend a ten-day course of either penicillin or amoxicillin. These antibiotics are inexpensive, narrow-spectrum, and have low rates of side effects. For people who are allergic to these drugs the IDSA suggests prescribing azithromycin for five days, first generation cephalosporin for ten days, or clindamycin or clarithromycin for ten days (Shulman et al., 2012). CDC/AAFP/ACP-ASIM guidelines recommend the use of erythromycin for patients allergic to penicillin (Cooper et al., 2001).

Whether and when patients should be treated with antibiotics for pharyngitis remains a controversial question. A perspective prevalent in Europe is that GABHS is a self-limiting disease with low rates of complication. Therefore, antibiotics are unnecessary. Some physicians believe that antibiotics use should be restricted because their benefits are either nonexistent or modest (Bisno, 2003; Group et al., 2012). Centor argues that empirical treatment of individuals with Centor scores of three or four is appropriate (Centor, 2012), although other studies assert that empirical treatment based on clinical decision rules leads to overuse of antibiotics (Humair et al., 2006; Shaikh et al., 2012). Limiting antibiotics to patients with a positive RADT or throat culture is another option (Neuner et al., 2003; Humair et al., 2006).

Disagreements about whether and when to prescribe antibiotics for pharyngitis exist because there is no clearly superior management strategy. As such, it is not surprising that different treatment strategies are advocated by physicians and guidelines, even within the same medical institution (Singh et al., 2006). One opinion is that the question of the best method to diagnose and treat GABHS doesn’t really matter. According to this view, all approaches besides empirical treatment have similar effectiveness and cost when the percentage of adults with pharyngitis who are GABHS-positive is approximately 10% (Neuner et al., 2003). Another reason for the lack of consensus on how best to diagnose and treat patients who may have GABHS are differences in how treatment goals are prioritized. Assumptions about the proportion of individuals with pharyngitis who are infected with GABHS also affect preferred management strategies (Singh et al., 2006).

The absence of a superior treatment standard suggests that greater emphasis should be placed on how physicians and pharmacists dispense antibiotics in practice, and how patients view the care they receive. Antibiotic prescription rates far exceed the proportion of GABHS-positive individuals with pharyngitis, indicating that physicians do not adhere to advice offered in clinical guidelines (McIsaac and Goel, 1997). How to overcome physicians’ inclination to prescribe antibiotics for GABHS is an important question that deserves attention. A study that compared antibiotic use in the United Kingdom and Holland found that British physicians prescribed antibiotics at twice the rate of their Dutch counterparts (Butler and Francis, 2008). Differences in medical education and the structure of the countries’ health systems may explain part of this variation. A better understanding of these factors could inform policy efforts to bring antibiotic prescription rates for patients with pharyngitis closer to the percentage of the population that is infected. Another approach is to focus on the role that pharmacists play in diagnosing and treating GABHS. Studies have found that training programs for community pharmacists have reduced levels of inappropriate antibiotic use while improving patient satisfaction (Demoré et al., 2018; Essack et al., 2018)

Treatment for pharyngitis also depends on patient expectations (Cooper et al., 2001). Individuals with sore throat often have strong opinions about whether they should take an antibiotic (Neuner et al., 2003). Physicians may overestimate patients’ desire for antibiotics, a misperception that contributes to excessive use of antibiotics (Kumar et al., 2003). What matters for many people in evaluating their treatment is whether their doctor seeks to understand their concerns (Cooper et al., 2001; Neuner et al., 2003). In many cases the relationship with a patient can be more effectively maintained by showing care than by writing a prescription for antibiotics (Kumar et al., 2003). Good communication skills are also important. Physicians need to be able to explain the benefits and risks of antibiotics to their patients in a clear manner (Butler and Francis, 2008; Tan et al., 2008). In sum, the quality of the doctor-patient relationship and interaction may be just as important as the particular diagnostic and treatment methods used for pharyngitis.

## Conclusion

Strep throat is a common infection that affects millions of adults and children annually. GABHS can be treated effectively with narrow-spectrum antibiotics. However, significant disagreements remain over how best to diagnose and treat the disease. The lack of consensus in the medical community is reflected in the guidelines provided by different professional associations in the United States and in Europe. These guidelines recommend a variety of approaches to managing GABHS. Therefore, physicians have wide discretion in determining how best to treat patients who present with pharyngitis. We conclude that major questions about the diagnosis and treatment of GABHS remain. Given the high incidence of strep throat and the possibility of complications, efforts should be made to resolve these ambiguities. Variations in approaches to the disease offered in clinical guidelines from influential professional associations are striking. Harmonizing these guidelines should be a priority for future research.

## Author Contributions

ZM and MG contributed to the literature search of the topic, design of the paper, and the writing of the manuscript. All authors contributed to the article and approved the submitted version.

## Conflict of Interest

The authors declare that the research was conducted in the absence of any commercial or financial relationships that could be construed as a potential conflict of interest.
